# Supplementary figures and images for: Building extraction from remote sensing imagery using SegFormer with post-processing optimization
Source: PLoS One. 2025 Dec 8;20(12):e0338104. doi: 10.1371/journal.pone.0338104 (PMC12685217; doi:10.1371/journal.pone.0338104)

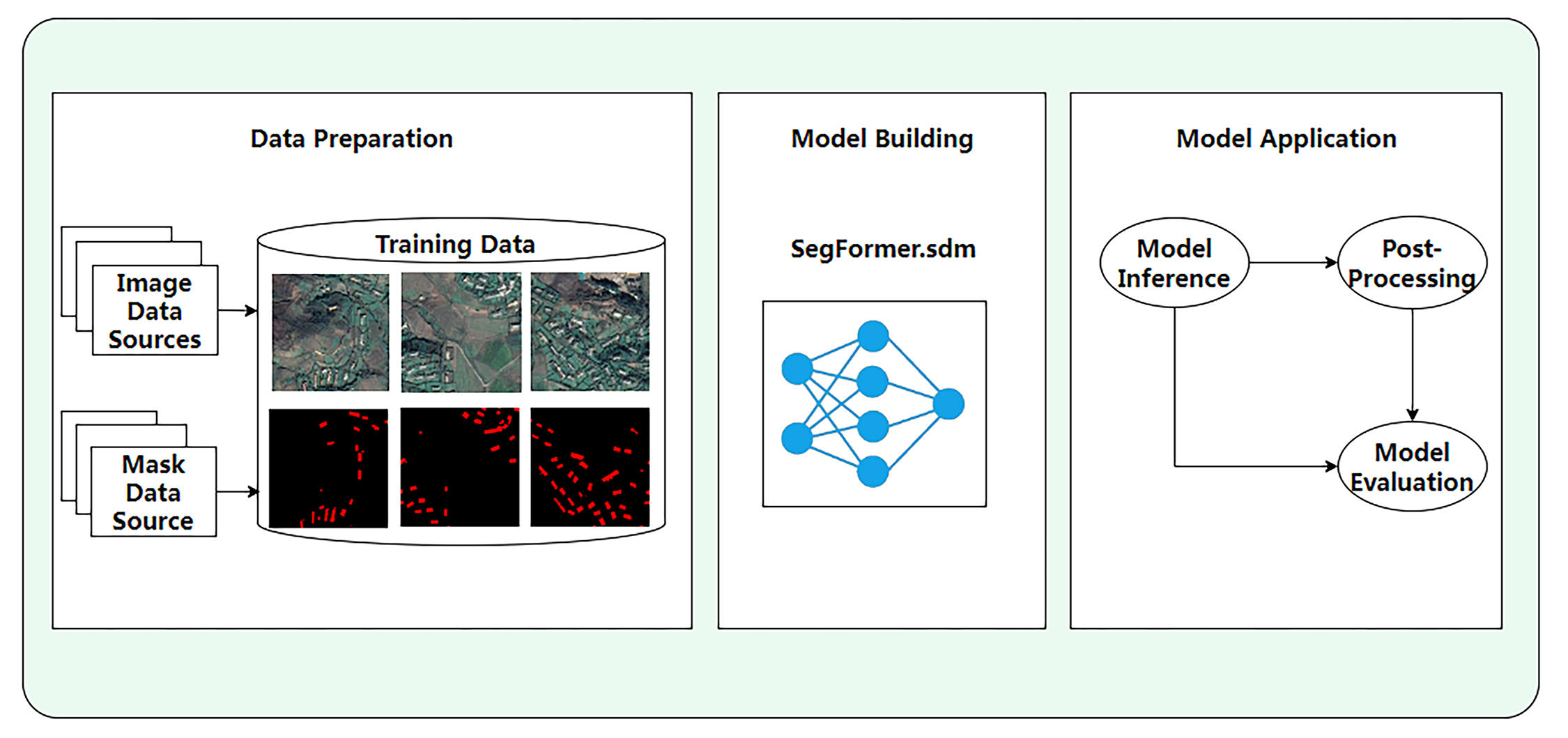

Supplement: S1 Fig — (TIF) [file pone.0338104.s001.tif]

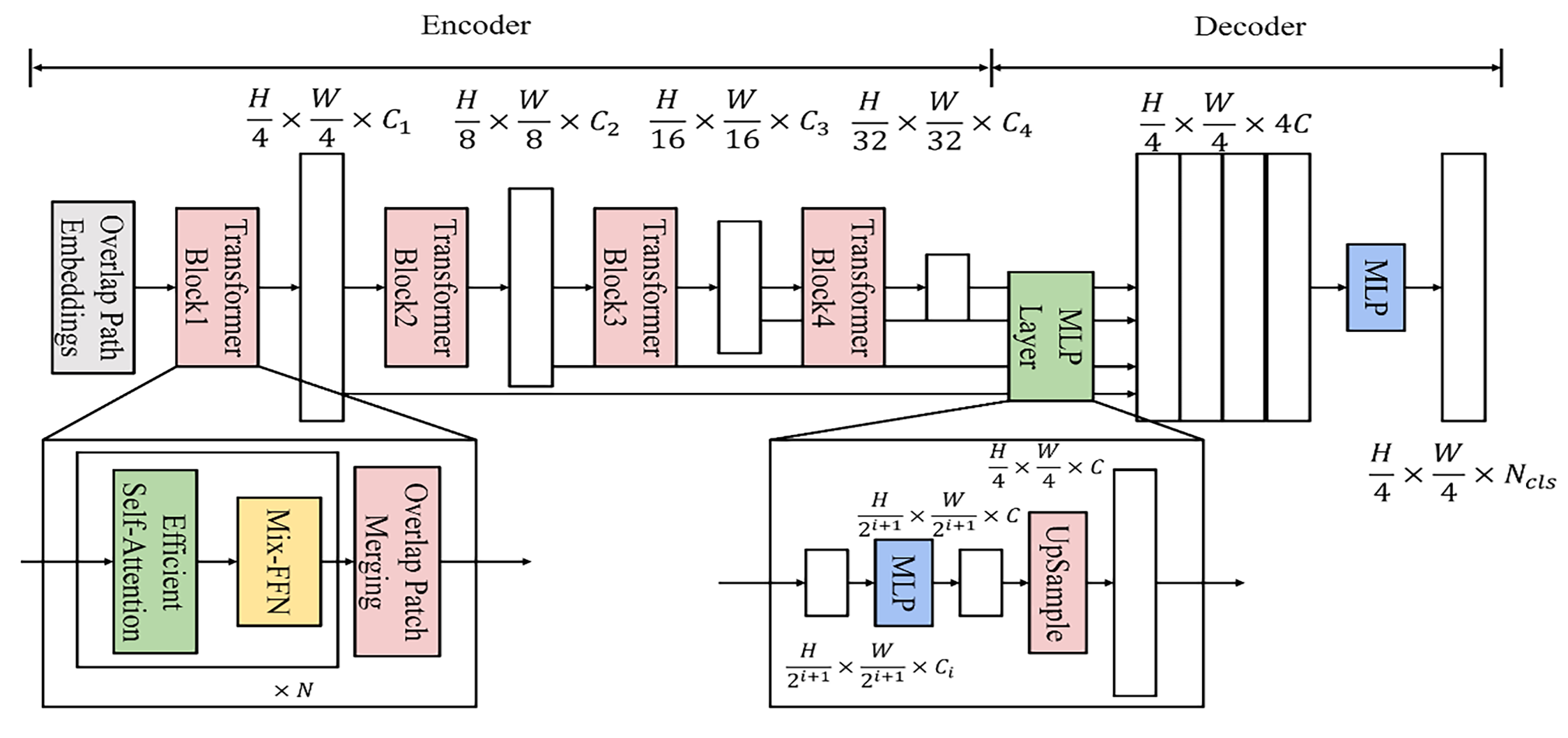

Supplement: S2 Fig — (TIF) [file pone.0338104.s002.tif]

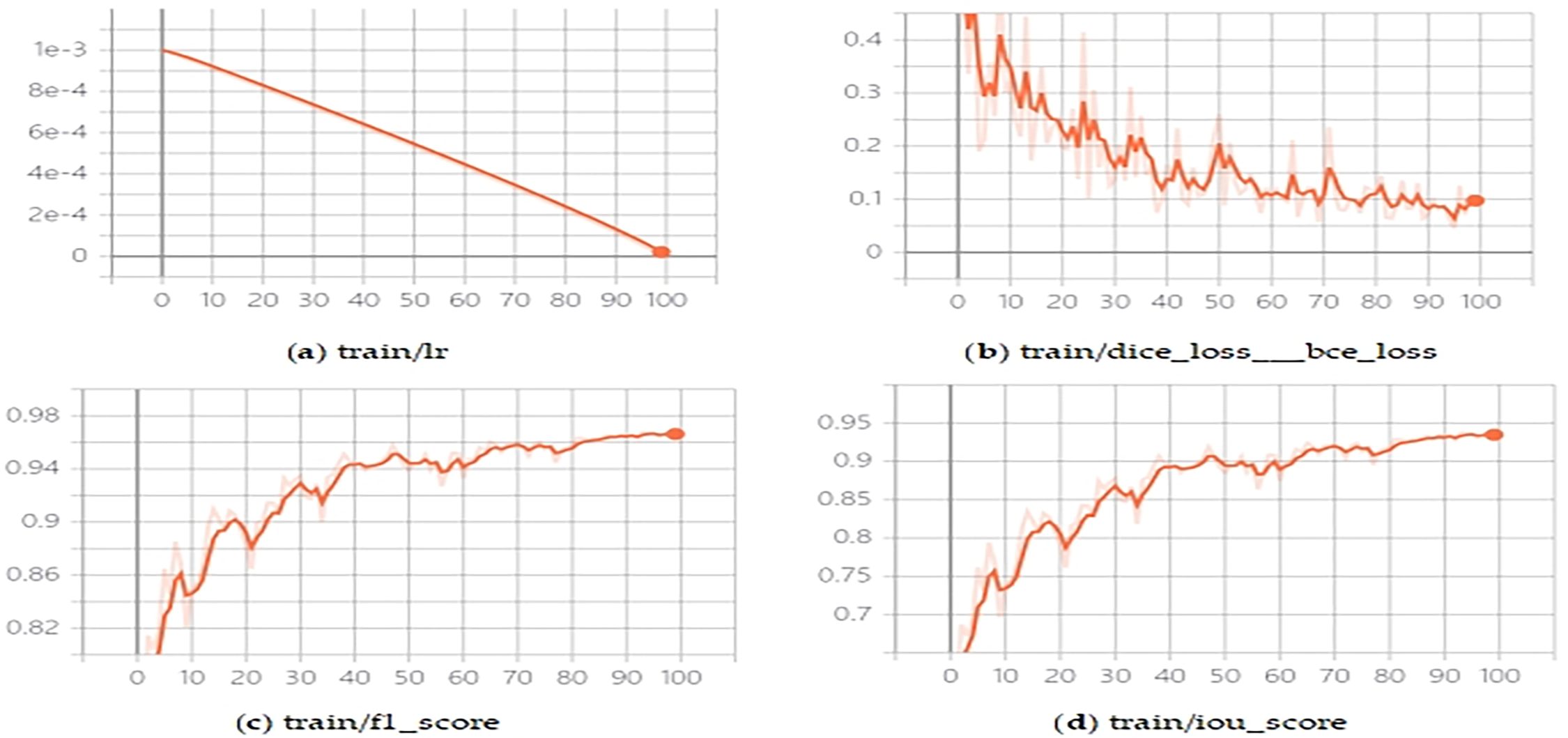

Supplement: S3 Fig — (a) Train/LR; (b) Train/dice_loss_BCE_loss; (c) Train/F1_score; (d) Train/IoU_score. (TIF) [file pone.0338104.s003.tif]

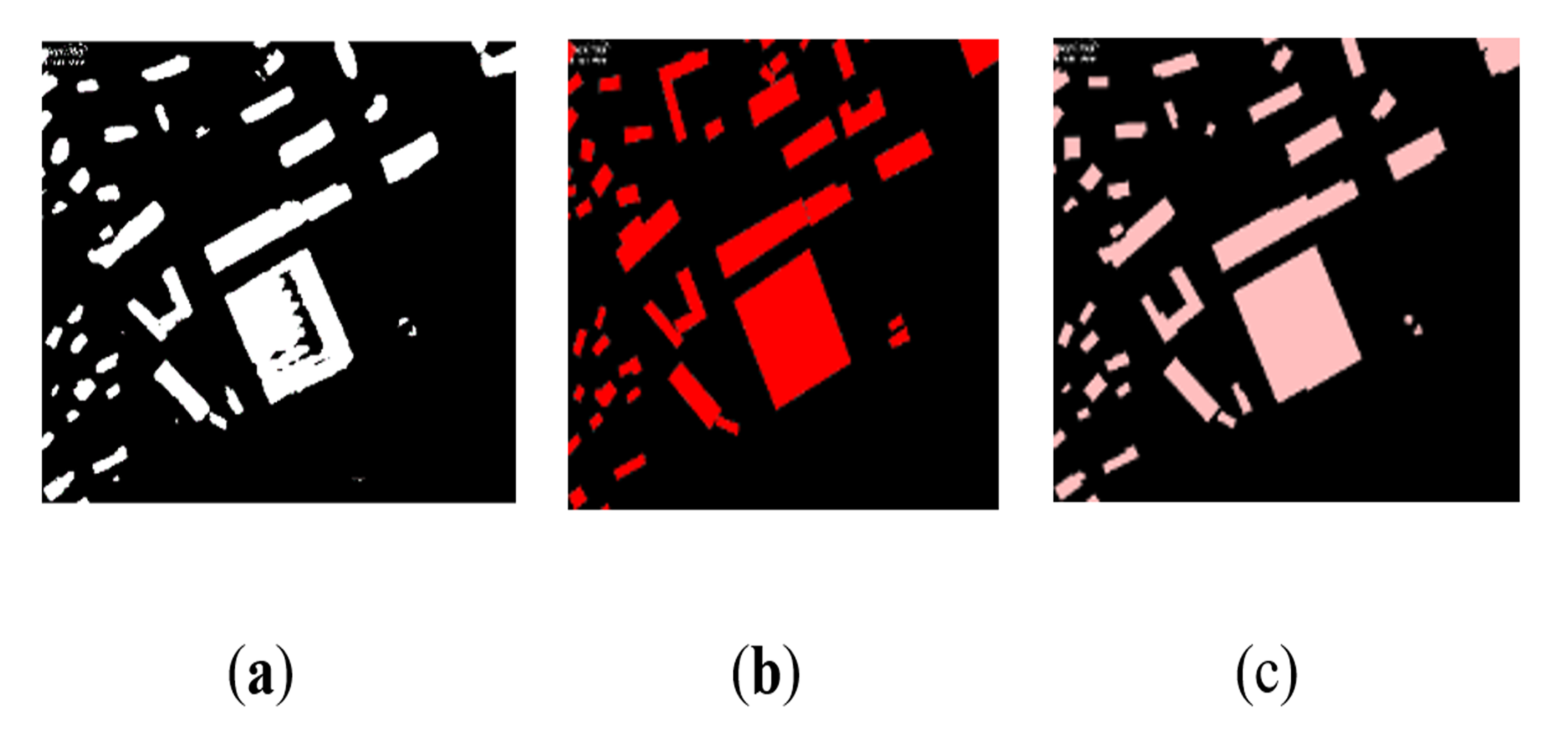

Supplement: S4 Fig — (a) Inferred results; (b) Validated label; (c) Optimised results. (TIF) [file pone.0338104.s004.tif]

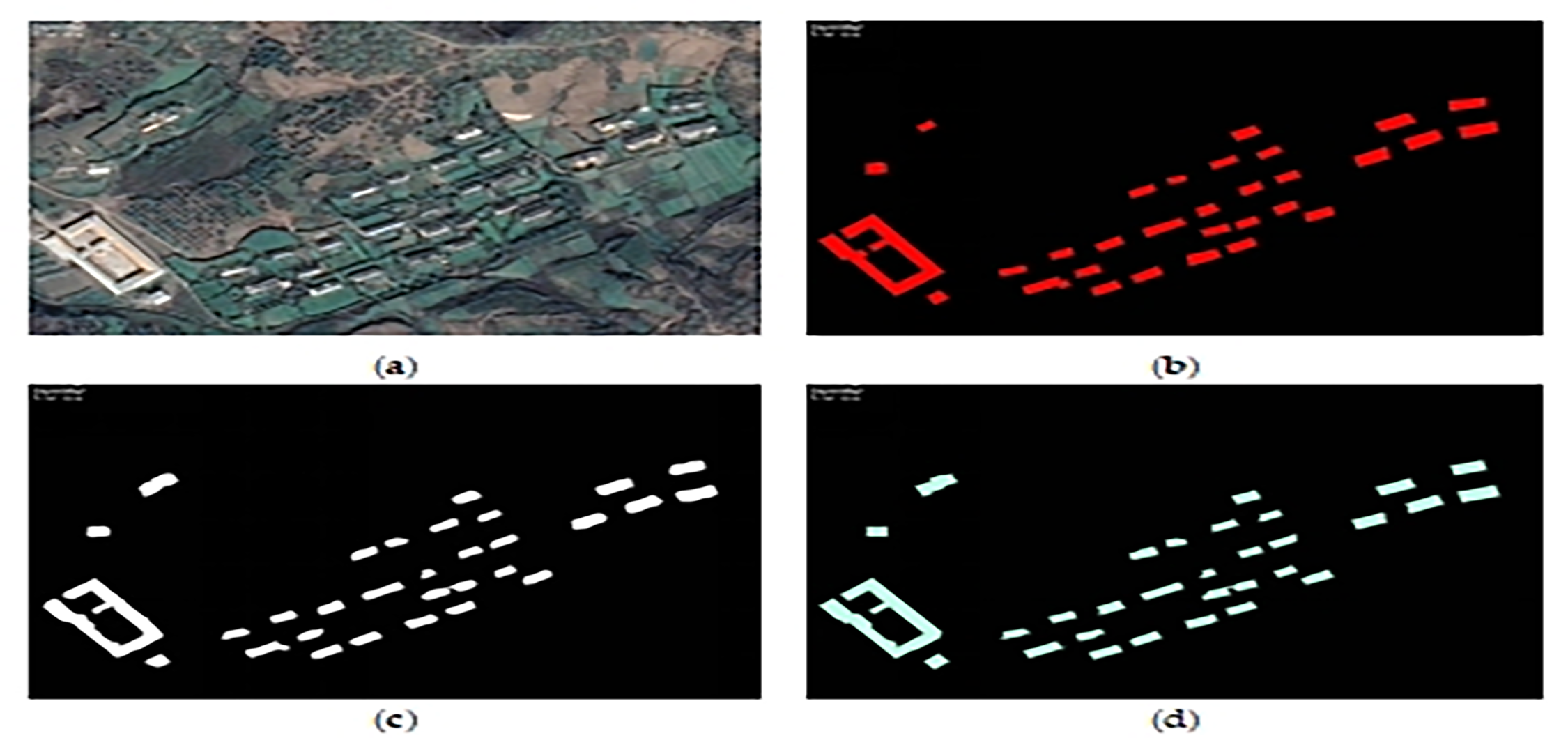

Supplement: S5 Fig — (a) Raw image; (b) Validated label; (c) Inferred results; (d) Optimised results. (TIF) [file pone.0338104.s005.tif]

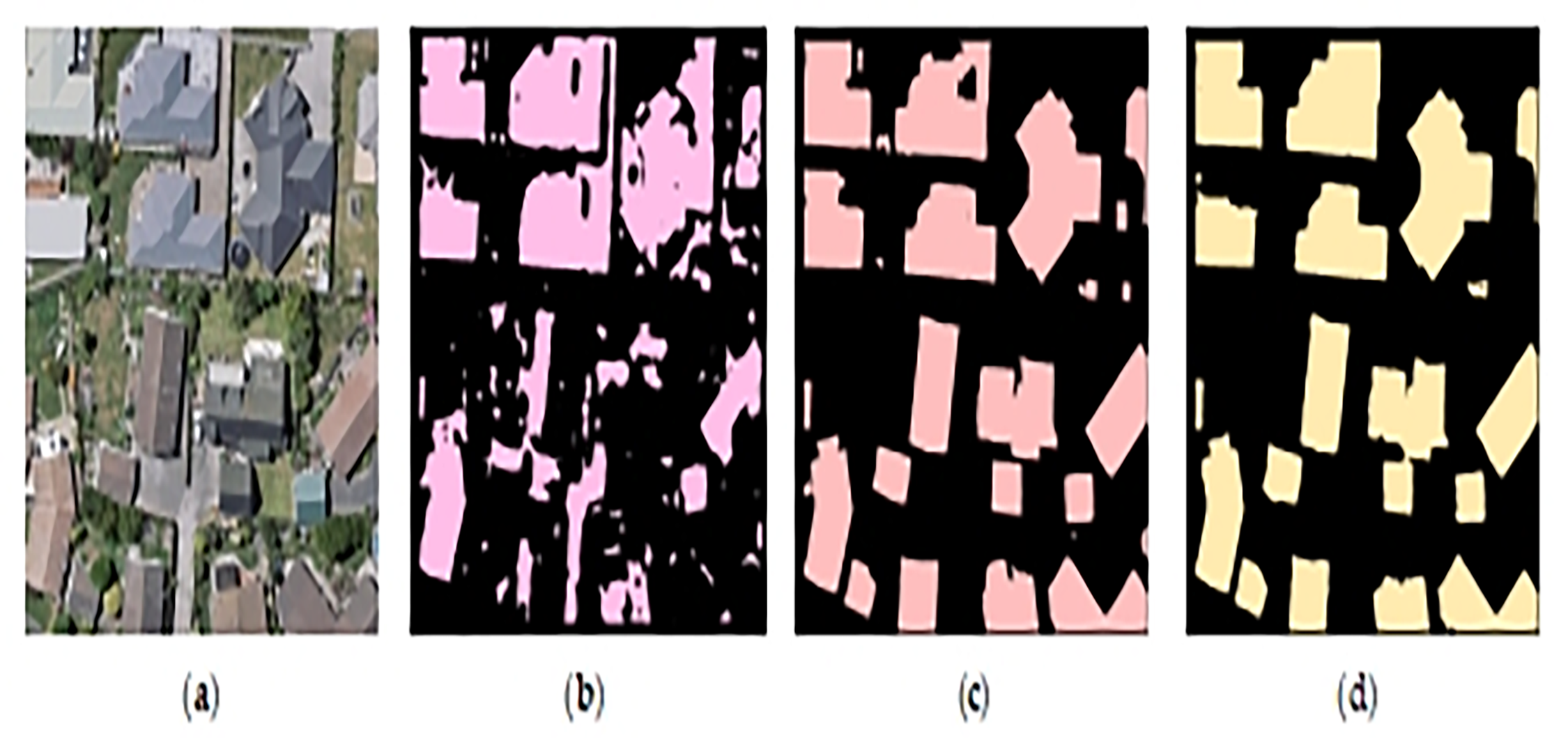

Supplement: S6 Fig — (a) Raw image; (b) DeeplabV3 + ; (c) SegFormer(Mit-B3); (d) SegFormer(Mit-B5). (TIF) [file pone.0338104.s006.tif]

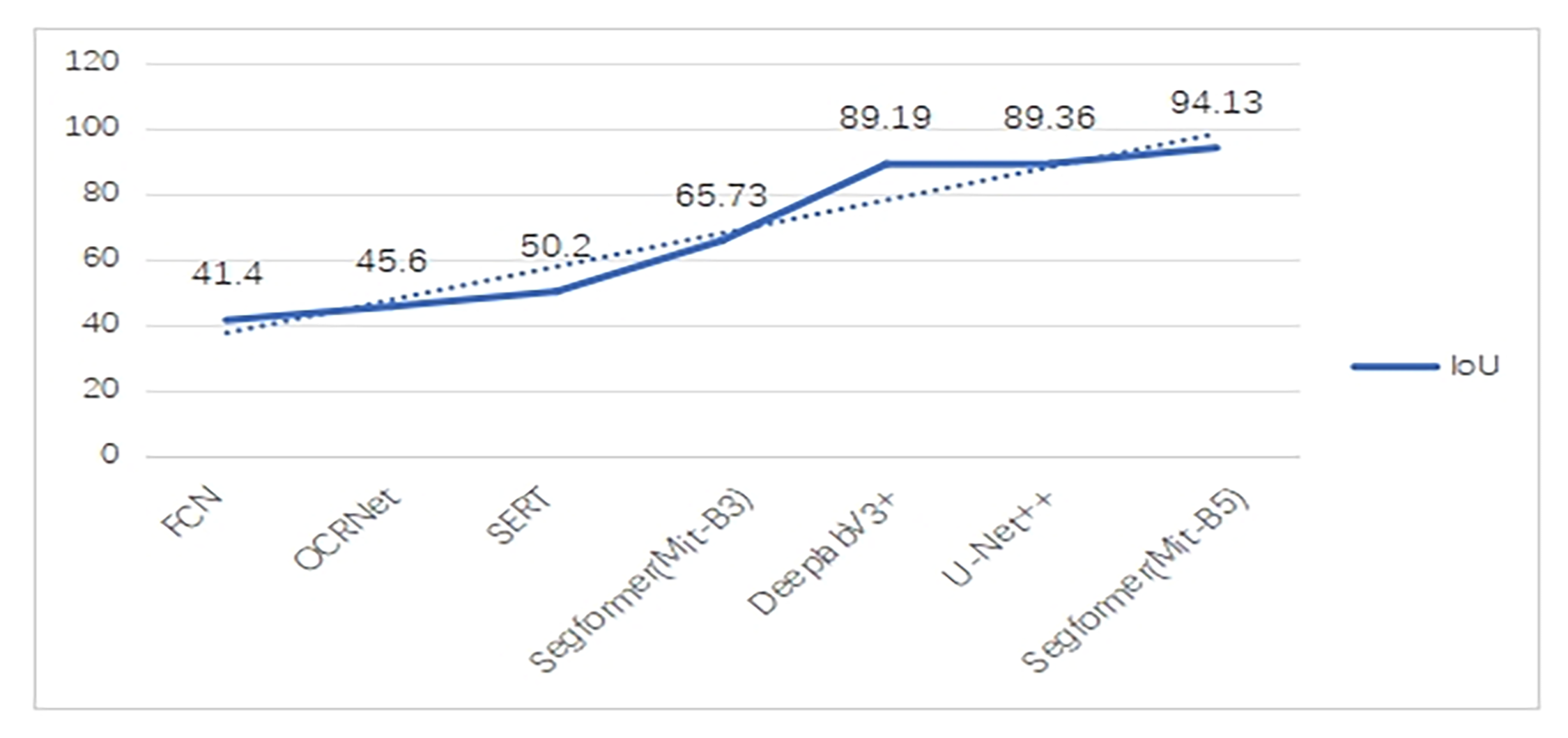

Supplement: S7 Fig — (TIF) [file pone.0338104.s007.tif]
