# Supplementary material for: Building extraction from remote sensing imagery using SegFormer with post-processing optimization
Source: PLoS One. 2025 Dec 8;20(12):e0338104. doi: 10.1371/journal.pone.0338104 (PMC12685217; doi:10.1371/journal.pone.0338104)
Supplement: S1 Table — (DOC) [file pone.0338104.s008.doc]

**Table 1. Datasets used in this paper.**

| **Dataset** | **Category** | **Building Number** | **Resolution** |
| --- | --- | --- | --- |
| WHU | train | 25749 | 0.45 m |
|  | Val | 34085 |  |
|  | test | 8358 | 0.45 m |
