# Supplementary material for: Building extraction from remote sensing imagery using SegFormer with post-processing optimization
Source: PLoS One. 2025 Dec 8;20(12):e0338104. doi: 10.1371/journal.pone.0338104 (PMC12685217; doi:10.1371/journal.pone.0338104)
Supplement: S2 Table — (DOC) [file pone.0338104.s009.doc]

**Table 2. Training configuration of the dataset.**

| **Item** | **WHU** |
| --- | --- |
| Train size | 1024 × 1024 |
| Epoch | 100 |
| Model | SegFormer |
| Learning rate | 0.001 |
| Backbone network | Mit-b3、Mit-b5 |
| Training log path | E:\log |
