# Supplementary material for: Building extraction from remote sensing imagery using SegFormer with post-processing optimization
Source: PLoS One. 2025 Dec 8;20(12):e0338104. doi: 10.1371/journal.pone.0338104 (PMC12685217; doi:10.1371/journal.pone.0338104)
Supplement: S3 Table — (DOC) [file pone.0338104.s010.doc]

**Table 3. Model evaluation results.**

| **Methods** | **BackBone** | **Data** | **IoU** | **Precision** | **F1** | **Recall** |
| --- | --- | --- | --- | --- | --- | --- |
| FCN [6] | ResNet-101 | ADE20K | 41.4 | - | - | - |
| OCRNet [6] | HRNet-W48 | ADE20K | 45.6 | - | - | - |
| SegFormer [6] | Mit-B3(S1-4) | ADE20K | 48.6 | - | - | - |
| SETR [6] | ViT-Large | ADE20K | 50.2 | - | - | - |
| SegFormer(Ours) | Mit-B3 | WHU | 65.73 | 80.12 | 79.32 | - |
| SegFormer [9] | Transconv | WHU | 74.23 | 87.35 | 84.27 | 83.61 |
| DeeplabV3+ [41] | - | WHU | 89.19 | 94.95 | 94.29 | 93.64 |
| U-Net++ [41] | - | WHU | 89.36 | 95.34 | 94.38 | 93.44 |
| SegFormer(Ours) | Mit-B5 | WHU | 94.13 | 96.70 | 96.58 | - |
