# Supplementary material for: Building extraction from remote sensing imagery using SegFormer with post-processing optimization
Source: PLoS One. 2025 Dec 8;20(12):e0338104. doi: 10.1371/journal.pone.0338104 (PMC12685217; doi:10.1371/journal.pone.0338104)
Supplement: Attachment — Submitted filename: Response_to_Reviewers-Round 1.doc [file pone.0338104.s013.doc]

**Response to Editors and Reviewers**

**Manuscript ID:** **[PONE-D-25-26895R1] - [EMID:89e8750006dca458]**
**Title:** Building Extraction from Remote Sensing Imagery Using SegFormer with Post-Processing Optimization
**Journal:** PLOS ONE
**Corresponding Author:** wanghaokun@bcnu.edu.cn

Dear Aiqing Fang and Reviewers,

We sincerely thank the Academic Editor for the constructive feedback and for giving us the opportunity to revise our manuscript. We have meticulously addressed all the points raised. The specific concerns regarding formatting, references, and punctuation have been thoroughly corrected throughout the entire manuscript. Our point-by-point responses are detailed below.

### ****Editor’s Comments****

1. **Comment:** References are not up-to-date.

**Response:** We thank the editor for this important comment. We have now comprehensively updated the reference list. Several outdated references have been replaced with more recent and relevant literature from the past 3-5 years to ensure the manuscript reflects the current state of the field. The entire reference list has also been reformatted to strictly adhere to the PLOS ONE citation style.

2. **Comment:** There are significant formatting issues with the entire text.

**Response:** Thank you for your feedback. We have carefully revised our manuscript in accordance with PLOS ONE's style requirements, using the provided templates:

<https://journals.plos.org/plosone/s/file?id=wjVg/PLOSOne_formatting_sample_main_body.pdf> and <https://journals.plos.org/plosone/s/file?id=ba62/PLOSOne_formatting_sample_title_authors_affiliations.pdf.>

The revisions encompass comprehensive formatting corrections, including adjustments to font type and size, line spacing, margin alignment, section headings, as well as the proper placement and captioning of figures and tables. Consistency in the use of italics and bolding has also been ensured throughout the manuscript. We have further verified that the file naming and structure comply with the journal's guidelines. We believe the manuscript now fully meets the required standards and thank you for your guidance.

3. **Comment:** There are numerous issues with punctuation consistency.

**Response:**  **We have performed a thorough, line-by-line proofreading of the entire manuscript to correct all inconsistencies in punctuation. This includes standardizing the use of commas, periods, colons, and semicolons, as well as ensuring correct and consistent use of spacing after punctuation marks according to standard English and journal conventions.**

**Once again, we are grateful for the editor's guidance. We believe the revisions have significantly improved the quality and presentation of our manuscript, and we hope it now meets the high standards of PLOS ONE.**

### ****Reviewer #1****

1. **Comment:** Requested to revise the abstract into a single paragraph format.

**Response:** Thank you for your feedback. We will revise the abstract into a single paragraph as requested to meet the journal's formatting guidelines.

### ****Reviewer #3****

1. **Comment:** All comments have been addressed, and the manuscript is recommended for acceptance.

**Response:** We sincerely appreciate the reviewer's positive assessment and recommendation for acceptance
